# Physical activity levels among Canadians using a health equity lens

**DOI:** 10.24095/hpcdp.46.2.01

**Published:** 2026-02

**Authors:** Justin J. Lang, Sarah E. Turner, Natalie Doan, Iryna Demchenko, Stephanie A. Prince, Karen C. Roberts, Marisol T. Betancourt, Rachel Colley, Rabina Jahan, Ashley Amson, Jean-Philippe Chaput

**Affiliations:** 1 Centre for Surveillance and Applied Research, Public Health Agency of Canada, Ottawa, Ontario, Canada; 2 School of Epidemiology and Public Health, Faculty of Medicine, University of Ottawa, Ottawa, Ontario, Canada; 3 Alliance for Research in Exercise, Nutrition and Activity (ARENA), University of South Australia, Adelaide, South Australia, Australia; 4 Healthy Active Living and Obesity Research Group, Children’s Hospital of Eastern Ontario Research Institute, Ottawa, Ontario, Canada; 5 Department of Community Health Sciences, University of Manitoba, Winnipeg, Manitoba, Canada; 6 School of Public Health Sciences, Faculty of Health, University of Waterloo, Ontario, Canada; 7 Department of Health Sciences, Carleton University, Ottawa, Ontario, Canada; 8 Health Analysis Division, Statistics Canada, Ottawa, Ontario, Canada; 9 Health Equity Policy Directorate, Public Health Agency of Canada, Ottawa, Ontario, Canada; 10 Policy and Planning, Canadian Heritage - Sport Canada, Ottawa, Ontario, Canada; 11 Interdisciplinary School of Health Sciences, University of Ottawa, Ottawa, Ontario, Canada; 12 Department of Pediatrics, Faculty of Medicine, University of Ottawa, Ottawa, Ontario, Canada

**Keywords:** physical activity, adult, youth, health equity, gender, socioeconomic position, Canada

## Abstract

**Introduction:**

This study examined physical activity (PA) levels among youth (12–17years) and adults (18 years and older) living in Canada by subgroups including gender, sexual orientation, population groups, education, and income.

**Methods:**

Data from the 2021 Canadian Community Health Survey (N = 44 239), a large national, cross-sectional survey, was used to examine self-reported daily PA time spent in active transportation, recreation, school/camp, occupational/household, and adherence to PA recommendations (≥ 60 minutes/day and ≥ 150 minutes/week of moderate-to-vigorous intensity PA for youth and adults, respectively) by population subgroups. Significant differences within subgroups were assessed with chi-square and Tukey-Kramer analyses.

**Results:**

Among youth, boys were more likely to meet the PA recommendation than girls (54.9% vs. 36.5%). Boys engaged in more recreational (36.0 vs. 24.0 min/day) and school/camp (24.0 vs. 15.9 min/day) PA than girls. Youth from households in the highest income quintile reported more recreational PA compared to those in the lowest income quintile (35.8 vs. 22.1 min/day). Among adults, there was a significant gender difference in PA recommendation adherence (women: 51.7% vs. men: 57.4%). Men engaged in more recreational (18.0 vs. 15.1 min/day) and occupational/household (26.4 vs. 15.4 min/day) PA than women. Recreational PA was significantly higher in households with the highest income (22.8 min/day) and education (17.4 min/day) compared to lowest income (10.4 min/day) and education (6.9 min/day), respectively. Few subgroup differences were observed for active transportation.

**Conclusion:**

PA inequalities persist in Canada. Future research should explore why these inequalities exist to help inform interventions.

**Highlights**

- Physical inactivity is an important modifiable risk factor for chronic disease. Identifying inequalities in participation can help guide equitable policies and interventions.
- We found significant inequalities in physical activity among youth and adults living in Canada.
- The largest inequalities were seen across income and education groups, with the more advantaged groups reporting significantly more physical activity across domains when compared to less advantaged groups.
- Active transportation did not differ significantly across population subgroups, suggesting an opportunity to equitably improve population physical activity levels.

## Introduction

Physical activity (PA) is strongly associated with health and well-being.^1^,^2^ It is recommended that youth participate in at least 60 minutes of moderate-to-vigorous intensity PA (MVPA) per day to gain optimal health benefits.^3^ For adults, the recommendation is at least 150 minutes of MVPA per week.^4^ Although PA is important to maintain or improve health, less than half of Canadian youth (43.9%) and adults (49.2%) meet PA recommendations. 5 PA occurs in a variety of contexts (e.g. transportation, recreation, school/camp, occupational/household), described as domains, which provide additional information about adherence not always apparent when looking at recommendation adherence alone. A detailed analysis of PA levels by domain, both within and between population subgroups, could better inform future interventions and policy development.

Not all population subgroups achieve the same levels of PA, due in part to the many social, economic and environmental factors that are recognized as key drivers of health outcomes, making it an important behavioural target in improving health equity. The first step in applying a health equity lens—a strategic and intentional approach to examining disparities in the achievement of an outcome across underserved and historically marginalized communities and population subgroups—is critical to generating the necessary evidence to address equity issues.^6^,^7^ This paper seeks to help identify certain population subgroups that are not achieving adequate PA for health.^8^

Among youth, several studies have explored disparities in sports participation.^9^ For instance, some American studies have found lower parent- or self-reported participation rates among youth from lower socioeconomic households and “minority” groups.^10^–^13^ Canadian data show that gender-diverse and sexual minority youth report less sports participation, with sexual minority youth engaging in significantly lower total PA than their heterosexual peers.^14^

Among adults, several studies have examined PA equity/equality and diversity across population subgroups. In Sweden and Chile, male adults reported higher levels of PA compared to females, while in Germany, no sex differences were observed in self-reported PA.^15^-^17^ Additionally, a large European study reported that higher education was negatively associated with meeting the PA recommendation among men but not women.^18^ However, in Australia, no significant PA differences were observed between education groups.^19^ In contrast, a Canadian study using accelerometer data showed that adults with the highest education and income levels were more likely to meet the PA recommendation than those with the lowest levels.^20^

It is evident that globally, inequalities in PA exist across socio-demographic groups.^9^–^18^ Examining PA levels through a health equity lens is essential for providing the evidence necessary to promote fairness, social justice and inclusivity in health promotion efforts to ensure equal opportunity to lead active and healthy lives. The Canadian Health Inequalities Data Tool provides interactive breakdowns and measures of inequalities for self-reported PA across factors, such as education, income, immigration status, living arrangement, and language spoken at home.^21^ However, the tool only reports on a single PA measure, i.e. meeting the PA recommendations, leaving room to expand by looking at health equity across domains of PA (e.g. school, work, leisure, active transportation). The Physical Activity, Sedentary Behaviour and Sleep (PASS) Indicators describe PA and 24-hour movement behaviours across a variety of population subgroups using different data sources,^22^ but do not explore differences by less studied subgroups such as sexual orientation, language, and living arrangements. We aimed to examine adherence to the PA recommendation, reporting no PA, as well as domain-specific PA across population subgroups such as gender, population group (i.e. race/ethnicity/visible minorities), Indigenous identity, immigration status, language spoken at home, household income, highest household education, rural/urban geography, living arrangement, sexual orientation (adults only), age group (adults only), living with chronic condition(s) (adults only), and employment status (adults only) for both youth aged 12 to 17 years and adults aged 18 years and older in Canada.

## Methods

***Data source***

We used national self-reported data from the 2021 Canadian Community Health Survey (CCHS), an ongoing cross-sectional survey that focusses on health status, health care utilization, and health determinants.^23^ The CCHS sampling frame included individuals aged 12 years and older. Excluded individuals were those living on First Nation Reserves, on Crown Land, in institutions, full-time members of the Canadian Armed Forces, youth aged 12-17 years living in foster homes, or persons living in the Quebec health regions of Nunavik and Terres-Cris-de-la-Baie-James, which accounted for less than 3% of the Canadian population. Due to these exclusions, the Indigenous identifying subpopulation had a low sample size and was not representative. 

The 2021 cycle took place between January 4, 2021, and January 31, 2022, andwas conducted over the phone using computer assisted interviews by trained Statistics Canada staff. The CCHS total response rate was 24.1% (adults = 23.8%, youth = 28.4%).^23^

Participants living in the three territories were excluded from this analysis because multiple cycles (i.e. 2021 and 2022) are needed to generate a large enough sample size to include territorial data. Participants who reported a daily average of more than 600 minutes of PA (10 hours) in any of the individual PA domains were considered outliers and their responses were also excluded (n_adolescents_=12; n_adults_=87). In total, 49 678 individuals provided responses; however, only 44 239 agreed to share their responses with partners such as the Public Health Agency of Canada (89.1% share rate). Of the 44 239 participants, 40 956 were adults (aged 18 years and older) and 3283 were youth (aged 12 to 17 years). This study used listwise deletion to maximize sample size across the different subgroups and PA measures.

Informed consent was obtained prior to the interview. Parent or guardian verbal permission was required to interview youth aged 12 to 14 years. The CCHS was approved by the Statistics Canada Office of Privacy Management and Information Coordination and the Data Ethics Secretariat. The CCHS can be used for research without additional research ethics board review (article 2.2 of the Tri-Council Policy Statement on Ethical Conduct for Research Involving Humans).^24^

***Physical activity measures***

We reported the amount of PA across four domains: active transportation, recreational PA, school or camp PA and occupational/household PA, described in detail below. Among youth, PA questions asked for daily amounts over each of the previous 7 days, whereas for adults, questions asked for a total amount for the previous 7 days. For youth, the daily amounts were summed and divided by 7 to generate a daily average except for school or camp PA where daily minutes were summed and divided by 5 (it was assumed that there was no school or camp on Saturday and Sunday). For adults, the total weekly amount was divided by 7 to generate a daily PA average for each domain. For all PA except active transportation, respondents were asked to consider activities that made them sweat at least a little and breathe harder to identify those of at least moderate intensity when reporting duration. For adults, participants were instructed to only report activities that lasted a minimum of 10 continuous minutes.

**Active transportation**

Youth participants were asked, “In the last 7 days, did you use active ways like walking or cycling to get to places such as school, the bus stop, work, the shopping centre, or to visit friends?” Adult participants were asked, “In the last 7 days, did you use active ways like walking or cycling to get to places such as work, school, the bus stop, the shopping centre or to visit friends?” Time spent in active transportation was reported as an average across all participants (i.e. values are low due to a high number of participants reporting 0 min of active transportation).

**Recreational PA**

Youth participants were asked, “In the last 7 days, did you do physical activities in your leisure time including exercising, playing an organized or non-organized sports or playing with your friends?” Adult participants were asked, “In the last 7 days, did you do sports, fitness or recreational physical activities, organized or non-organized, that lasted a minimum of 10 continuous minutes?”

**School or camp PA**

Youth participants were asked: “In the last 7 days, did you do sports, fitness or recreational physical activities while at school or day camp?” 

**Occupational or household PA**

Youth participants were asked: “In the last 7 days, did you do any other physical activities that you have not already reported, for example, while you were doing paid or unpaid work or helping your family with chores?” Adult participants were asked: “In the past 7 days, did you do any other physical activities while at work, in or around your home or while volunteering?” 

**PA recommendation adherence**

Adherence to the PA recommendation was derived by summing the total number of minutes participants engaged in active transportation and moderate-to-vigorous intensity recreation, school or camp (for youth only), and occupation or household PA. Adherence was defined as a daily average of ≥60 minutes for youth and ≥150minutes per week for adults, with bouts of a minimum of 10 minutes.^3^,^4^

**No MVPA reported**

Adult participants who reported zero moderate-to-vigorous intensity PA (MVPA) minutes were classified as having ‘no PA reported’. This was uncommon in youth and, as a result, it was not explored in this group. 

***Measure of population subgroups***

The PA data were stratified by nine population subgroup variables measured using similar questions among youth and adults. We selected subgroups for analysis based on their concordance with many of the subgroups identified in the Wheel of Privilege and Power.^25^ Participants were asked about their gender (man, woman, gender diverse). Population group was measured using subgroups defined by Statistics Canada,^26^ with some groupings collapsed to allow for reportable estimates: White, Black, East Asian (Chinese, Japanese, and Korean), Southeast Asian (including Filipino), South Asian, West Asian and Arab, Latin American, and another visible minority (including multiple visible minorities). Indigenous identity included First Nations, Mtis, and Inuk (Inuit) living off reserves or other Indigenous settlements. Immigration status was determined by asking participants if they are or have ever been a landed immigrant or non-permanent resident, with a follow-up question about the number of years since immigration (e.g. non-immigrant [Canadian-born], 5 years or less, 6 to 10 years, and greater than 10 years). Participants were asked about the language most often spoken at home and the data were categorized as: English only, French only, English and French, English and/or French and other language, or other language only. Pre-tax household income for the year was adjusted to the low-income cut-off relative to the household and community size, with groups divided by quintile (e.g. five groups with the highest quintile representing the highest income group). Highest level of household education was measured using these three categories: less than high school, high school with no post-secondary, and post-secondary certificate, diploma or university degree. Urban/rural geography included four categories: living in a rural area (less than 1000), small population centre (1000 to 29 999), medium population centre (30 000 to 99 999), or large urban population centre (≥ 100000). 

There were differences in the response options for the living arrangement variable between youth and adults. Among youth, living arrangement included the following categories: lives with two parents, or lives with a single parent (could include birth, step or adoptive parent). Among adults, living arrangement included the following categories: unattached living alone, living with spouse/partner with no children, parent living with spouse/partner and children, or single parent living with children. 

We also included the following four population subgroup variables for adults only. Sexual orientation was classified using the following response options: heterosexual, gay or lesbian, and bisexual or pansexual. Age was measured using the following categories: 18–34, 35–49, 50–64, 65–79, and 80 years and older. Participants were asked to self-report if they had any of the following long-term/chronic health conditions diagnosed by a health professional: diabetes, arthritis, heart disease, anxiety (e.g. phobia, obsessive-compulsive disorder) or depressive (e.g. depression, bipolar, mania, dysthymia) disorders, cancer, Alzheimer’s, or the effects of a stroke. A subgroup variable was created to indicate the presence of one of the listed chronic conditions, two or more listed chronic conditions (multimorbidity), or no chronic conditions.^27^ Finally, participants aged 18 to 75 years were asked to report on employment status by indicating, in the previous week, if they worked, were absent from a job, or if they were unemployed. Participants who reported being employed were stratified into part-time or full-time work schedule.

***Statistical analysis***

For all analyses, we included the sample and bootstrap weights provided by Statistics Canada to account for the complex survey design. We calculated the prevalence and 95% confidence intervals (CI) of each population subgroup variable for categorial PA measures (e.g. meeting PA recommendation and no PA reported) and means and 95% CI for continuous PA measures (e.g. minutes per day of active transportation, recreation PA, school or camp PA, and occupational or household PA). Group differences for categorical PA measures were assessed using chi-square analyses. Group differences for continuous PA measures were assessed using post-hoc Tukey-Kramer tests, adjusted for multiple comparisons, to determine significant differences within group means for continuous PA variables. For this post-hoc test we used an alpha of *p* ≤0.001 to identify highly significant between-group differences. Following Statistics Canada release guidelines, we suppressed estimates with high coefficients of variation (i.e. > 35%).^23^ All statistical analyses were conducted using SAS Enterprise Guide 7.1 (SAS Institute Inc., Cary, NC, USA).

## Results

***Youth physical activity***

Table 1 provides an overview of the differences in PA across population subgroups for youth. The percentage of girls (36.5%) meeting the PA recommendation was nearly 20 percentage points lower than that of boys (54.9%). Girls had significantly lower average daily minutes of recreation (24.0 vs. 36.0 minutes/day) and school or camp (15.9 vs. 24.0 minutes/day) PA compared to boys. There were no significant within group gender differences in active transportation or occupational or household PA. Participants who identified as South Asian reported the least amount of time spent in active transportation (12.1 minutes/day), and those who identified as Southeast Asian reported the lowest amount of school or camp PA (10.7 minutes/day). There were significant differences in meeting PA recommendations and school or camp PA within the Indigenous identity population subgroup. Compared to non-immigrants (i.e. Canadian-born), youth who had immigrated to Canada within the last 5 years reported significantly lower recreation PA (12.1 vs. 31.7 minutes/day). Youth in the lowest household income quintile reported significantly lower recreation PA compared to those in the highest income quintile (22.1 vs. 35.8 minutes/day). There were no significant differences by the language spoken most often at home, household education, rural/urban geography, and living arrangement.

**Table 1:** Differences in physical activity by population subgroups among youth aged 12 to 17 years in Canada

Characteristic	Unweighted sample size (row) range across all PA variables	Meeting PA recommendation (≥60 min/day) % (95%CI)	Active transportation (min/day) mean (95%CI)	Recreation PA (min/day) mean (95%CI)	School/camp PA (min/day) mean (95%CI)	Occupation/ household PA (min/day) mean (95%CI)
**Unweighted sample size (column) (n)**	N/A	3043	3160	3189	3234	3237
Gender						
Boys	1573–1674	54.9 (51.1–58.7)	25.3 (22.0–28.7)^a^	36.0 (32.6–39.4)^a^	24.0 (21.1–26.9)^a^	8.5 (6.6–10.4)^a^
Girls	1441–1539	36.5 (32.8–40.2)	20.9 (17.2–24.7)^a^	24.0 (20.5–27.5)^b^	15.9 (14.1–17.7)^b^	7.0 (5.3–8.7)^a^
Gender diverse	^c^	^F^	^F^	^F^	^F^	^F^
*p*-value	N/A	< 0.0001	N/A	N/A	N/A	N/A
Population group						
White	2118–2259	49.3 (45.9–52.6)	22.5 (20.2–24.7)^a^	31.6 (29.0–34.2)^a^	21.7 (19.3–24.1)^a^	8.2 (6.6–9.7)^a^
Black	119–124	33.3 (21.2–45.5)^E^	13.0 (7.9–18.1)^a^^b^^E^	24.8 (10.5–39.1)^a^^E^	18.4 (10.4–26.4)^a^^b^^E^	^F^
East Asian (Chinese, Japanese, Korean)	118–124	42.4 (29.7–55.0) ^E^	^F^	35.5 (14.1–56.9)^a^^E^	26.5 (14.1–56.9)^a^^b^^E^	^F^
Southeast Asian (including Filipino)	123–127	45.9 (33.0–58.7)	29.8 (18.3–41.2)^a^^b^^E^	27.5 (17.4–37.6)^a^^E^	10.7 (6.5–14.8)b,^E^	^F^
South Asian	128–133	34.6 (23.8–45.3)^E^	12.1 (8.1–16.2)b,^E^	27.6 (18.9–36.3)^a^^E^	21.2 (14.7–27.8)^a^^b^^E^	4.7 (2.0–7.3)^a^^E^
West Asian and Arab	101–110	29.9 (19.4–40.4) ^E^	17.9 (10.4–25.4)^a^^b^^E^	23.4 (16.4–30.5)^a^^E^	14.6 (7.5–21.6)^a^^b^^E^	^F^
Latin American	39–41	83.5 (69.3–97.8)	67.9 (34.4–101.3)^a^^b^^E^	46.4 (17.7–75.1)^a^^E^	^F^	^F^
Another visible minority (including multiple visible minorities)	28–31	^F^	15.8 (8.1–23.5)^E^	^F^	^F^	^F^
*p*-value	N/A	< 0.0001	N/A	N/A	N/A	N/A
Indigenous identity						
First Nations	122–134	32.7 (20.8–44.6)^E^	18.4 (10.1–26.7)^a^^E^	19.9 (11.0–28.8)^a^^E^	7.6 (4.0–11.2)^a^^E^	^F^
Mtis	106–117	63.8 (49.8–77.8)	38.5 (24.9–52.1)^a^^E^	26.0 (17.1–34.9)^a^^E^	23.5 (15.7–31.3)^b^^E^	^F^
Inuk (Inuit)	^d^	^F^	^F^	^F^	^F^	^F^
*p*-value	N/A	0.0003	N/A	N/A	N/A	N/A
Immigration						
Non-immigrant (Canadian-born)	2678–2853	48.4 (45.5–51.4)	24.1 (21.3–26.9)^a^	31.7 (29.0–34.5)^a^	20.6 (18.7–22.5)^a^	8.4 (6.9–9.9)^a^
5 years or less since immigration	116–120	23.2 (12.7–33.6)^E^	14.4 (9.1–19.7)^a^^E^	12.1 (7.9–16.3)^b^^E^	13.7 (8.2–19.3)^a^^E^	4.4 (1.5–7.3)^a^^b^^E^
6 to 10 years since immigration	98–104	40.4 (27.9–52.9)^E^	20.4 (12.0–28.8)^a^^E^	29.0 (20.5–37.6)^a^^b^^E^	20.5 (11.9–29.1)^a^^E^	2.7 (0.9–4.4)^b^^E^
More than 10 years since immigration	88–94	33.3 (20.3–46.3)^E^	18.3 (10.6–26.0)^a^^E^	24.8 (12.4–37.1)^a^^b^^E^	19.1 (9.6–28.6)^a^^E^	^F^
*p*-value	N/A	0.0003	N/A	N/A	N/A	N/A
Language spoken most often at home						
English only	1924–2039	47.8 (44.2–51.3)	22.2 (19.9–24.5)^a^	31.3 (28.4–34.1)^a^	21.4 (19.1–23.7)^a^	8.2 (6.6–9.8)^a^
French only	552–614	45.4 (39.4–51.4)	22.3 (17.2–27.3)^a^	27.6 (23.0–32.3)^a^	20.3 (15.1–25.5)^a^	6.0 (3.1–9.0)^a^^E^
English and French	107–114	54.5 (41.0–68.0)	23.1 (15.7–30.5)^a^^E^	31.7 (22.9–40.6)^a^	20.7 (12.0–29.4)^a^^E^	7.9 (3.6–12.3)^a^^E^
English and/or French, and other language	271–282	40.7 (32.5–48.9)	23.2 (15.4–31.0)^a^^E^	27.6 (20.4–34.8)^a^	15.6 (12.1–19.1)^a^	7.8 (3.4–12.3)^a^^E^
Other language only	184–194	41.2 (30.5–51.9)	33.2 (13.3–53.0)^a^^E^	31.4 (16.6–46.3)^a^^E^	15.8 (9.2–22.4)^a^^E^	7.9 (3.5–12.2)^a^^E^
*p*-value	N/A	0.3041	N/A	N/A	N/A	N/A
Household income						
Quintile 1 (lowest)	654–686	37.7 (32.4–43.0)	23.6 (18.7–28.4)^a^	22.1 (18.2–26.0)^a^	16.0 (12.7–19.2)^a^	8.3 (5.5–11.2)^a^^E^
Quintile 2	607–654	46.7 (40.6–52.8)	25.2 (19.9–30.4)^a^	31.8 (25.7–37.9)^a^^b^	17.4 (14.4–20.4)^a^	8.2 (5.1–11.3)^a^^E^
Quintile 3	633–678	42.8 (36.5–49.0)	22.6 (14.6–30.6)^a^^E^	33.6 (27.1–40.1)^a^^b^	17.5 (14.2–20.7)^a^	5.7 (4.1–7.3)^a^
Quintile 4	567–602	52.2 (46.0–58.5)	21.9 (17.8–26.0)^a^	30.8 (26.0–35.6)^a^^b^	22.3 (18.5–26.0)^a^	8.8 (5.4–12.2)^a^^E^
Quintile 5 (highest)	480–509	53.1 (46.3–59.8)	23.2 (18.9–27.5)^a^	35.8 (30.3–41.4)^b^	30.3 (23.2–37.3)^a^	7.4 (3.8–10.9)^a^^E^
*p*-value	N/A	0.0045	N/A	N/A	N/A	N/A
Highest household education						
Less than secondary school graduation	104–115	40.6 (25.8–55.4)	29.9 (15.0–44.8)^a^^E^	20.4 (7.7–33.0)^a^^E^	15.9 (5.1–26.7)^a^^E^	9.3 (2.9–15.6)^a^^E^
Secondary school graduation, no post-secondary education	313–338	50.4 (42.5–58.3)	27.0 (19.6–34.4)^a^	32.8 (22.1–43.5)^a^^E^	17.2 (13.5–21.0)^a^	10.4 (7.0–13.8)^a^^E^
Post-secondary certificate diploma or university degree	2564–2714	46.0 (42.9–49.1)	21.6 (19.5–23.7)^a^	30.0 (27.7–32.2)^a^	20.5 (18.5–22.4)^a^	7.4 (6.0–8.9)^a^
*p*-value	N/A	0.4885	N/A	N/A	N/A	N/A
Rural/urban geography						
Rural area (less than 1000)	883–934	53.0 (48.5–57.5)	21.3 (17.8–24.8)^a^	34.2 (29.9–38.6)^a^	23.5 (20.5–26.4)^a^	12.7 (9.3–16.2)^a^
Small population centre (1000 to 29 999)	624–673	49.1 (43.5–54.6)	23.0 (18.7–27.2)^a^	30.1 (26.1–34.2)^a^	20.7 (15.1–26.4)^a^	9.7 (6.6–12.8)^a^^E^
Medium population centre (30 000 to 99 999)	356–386	43.3 (36.2–50.3)	18.9 (14.6–23.1)^a^	28.2 (22.9–33.5)^a^	17.5 (13.3–21.6)^a^	7.6 (4.4–10.8)^a^^E^
Large urban population centre (≥100 000)	1180–1245	43.7 (39.7–47.7)	24.4 (20.6–28.2)^a^	29.4 (25.7–33.1)^a^	19.1 (16.7–21.4)^a^	5.9 (4.2–7.5)^a^
*p*-value	N/A	0.02	N/A	N/A	N/A	N/A
Living arrangement						
Lives with two parents	2155–2284	47.8 (44.5–51.1)	22.2 (19.8–24.6)^a^	31.3 (28.7–33.9)^a^	20.9 (18.8–23.0)^a^	8.2 (6.6–9.9)^a^
Lives with a single parent	566–613	42.7 (37.0–48.5)	23.5 (18.8–28.2)^a^	25.6 (20.5–30.7)^a^	17.2 (13.7–20.7)^a^	6.8 (4.4–9.3)^a^^E^
*p*-value	N/A	0.1561	N/A	N/A	N/A	N/A

**Source:** Canadian Community Health Survey, 2021.

**Abbreviations:** CI, confidence interval; CV, coefficient of variation; MVPA, moderate-to-vigorous intensity physical activity; PA, physical activity. 

**Notes:** *p*-values were calculated using chi-square tests. Means with different superscripts (^a^ or ^b^) indicate that the means were statistically significantly different at *p* ≤ 0.001 based on Tukey-Kramer post-hoc comparisons (i.e. the same superscript letter indicates that the means were not statistically different from each other). 

^c^ 25 individuals who reported gender diverse were excluded from the analysis. 

^d^ 12 individuals who identified as Inuit were excluded from the analysis.

^E^ There is high sampling variability associated with these estimates (i.e. 15 < CV ≤ 35). 

^F^ These estimates do not meet Statistics Canada’s quality standards and therefore cannot be reported (i.e. CV > 35).

***Adult physical activity***

Table 2 provides an overview of the differences in PA across population subgroups for adults. Women (51.7%) had a significantly lower percentage of meeting the PA recommendation compared to men (57.4%). Women (23.7%) also had a significantly higher percentage reporting no PA compared to men (21.1%). These gender differences in adherence were driven by significant differences in recreation PA (15.1 vs. 18.0 minutes/day) and occupation or household PA (15.4 vs. 26.4 minutes/day). Similar to youth, there were no significant gender differences in active transportation among adults. Among population groups, those who reported being East (11.7 minutes/day), South (11.8 minutes/day), and West Asian (10.7 minutes/day) had the lowest occupational or household PA levels. There were no significant within group differences in the Indigenous identity subgroup. Compared to non-immigrants, immigrants who had lived in Canada for more than 10 years reported significantly lower recreation PA (13.6 vs. 17.6 minutes/day) and occupation or household PA (15.5 vs. 23.3 minutes/day). Adults who spoke another language only (12.2 minutes/day) or French only (17.6 minutes/day) most often at home had significantly lower occupation or household PA compared to those who spoke English only (23.7 minutes/day). There was a significant household income and education gradient whereby higher levels were associated with greater recreation PA. Those in the highest household income (22.8 minutes/day) and education (17.4 minutes/day) subgroups reported more than double the amount of recreation PA compared to the lowest household income (10.4 minutes/day) and education (6.9 minutes/day) subgroups. Adults living in rural areas reported significantly more occupation/household PA compared to those in large urban population centres (29.9 vs. 17.5 minutes/day). There was also a significant age gradient where those aged 65–79 and 80 years and older reported significantly less PA compared to younger individuals across most PA domains. Adults with multimorbidity also reported significantly lower PA levels across all domains when compared to participants with no or one chronic condition. Lastly, adults who were unemployed reported significantly less occupation or household PA compared to their employed counterparts (13.2 vs. 27.8 minutes/day). 

**Table 2:** Differences in physical activity by population subgroups among adults aged 18 years and older in Canada

Characteristic	Unweighted sample size (row) range across all PA variables	Meeting PA recommendation (≥150 min/week) % (95%CI)	No MVPA reported % (95%CI)	Active transportation (min/day) mean (95%CI)	Recreation PA (min/day) mean (95%CI)	Occupation/ household PA (min/day) mean (95%CI)
**Unweighted sample size (column) (n)**	N/A	40 430	40 430	40 747	40 790	40 611
Gender						
Men	18 084–18 248	57.4 (56.1–58.6)	21.1 (20.0–22.1)	14.2 (13.1–15.2)^a^	18.0 (17.1–18.8)^a^	26.4 (24.8–28.0)^a^
Women	22 283–22 476	51.7 (50.5–52.9)	23.7 (22.8–24.7)	12.4 (11.6–13.2)^a^	15.1 (14.1–16.1)^b^	15.4 (14.2–16.5)^b^
Gender diverse	47	^F^	^F^	^F^	10.6 (4.1–17.1)^a^^b^^E^	^F^
*p*-value	N/A	< 0.0001	0.0012	N/A	N/A	N/A
Population group						
White	34 413–34 859	57.0 (56.1–57.8)	21.5 (20.8–22.2)	13.3 (12.6–14.0)^a^	17.4 (16.7–18.0)^a^	22.6 (21.5–23.7)^a^
Black	624–634	46.4 (40.0–52.7)	23.1 (17.8–28.4)	12.1 (9.1–15.2)^a^	11.6 (9.0–14.2)^a^	16.7 (11.2–22.2)^a^^b^^E^
East Asian (Chinese, Japanese, Korean)	1067–1077	44.3 (39.6–48.9)	24.3 (20.3–28.2)	10.1 (8.0–12.1)^a^	16.8 (10.3–23.3)^a^^E^	11.7 (8.1–15.2)^b^^E^
Southeast Asian (including Filipino)	711–722	50.3 (44.3–56.3)	26.3 (21.0–31.7)	13.4 (10.0–16.9)^a^	12.0 (9.0–14.9)^a^	22.4 (14.5–30.4)^a^^b^^E^
South Asian	840–847	44.4 (39.2–49.6)	28.1 (23.5–32.7)	12.1 (8.9–15.3)^a^	13.0 (10.8–15.2)^a^	11.8 (7.7–16.0)^b^^E^
West Asian and Arab	341–345	44.0 (35.9–52.1)	23.8 (17.5–30.1)	13.9 (9.6–18.2)^a^^E^	11.1 (8.2–14.0)^a^	10.7 (5.1–16.3)^b^^E^
Latin American	253–255	54.9 (45.5–64.4)	19.3 (12.6–26.0)^E^	20.8 (9.5–32.1)^a^^E^	17.6 (11.7–23.4)^a^^E^	14.9 (6.2–23.7)^a^^b^^E^
Another visible minority (including multiple visible minorities)	159–160	48.3 (35.3–61.3)	28.2 (16.6–39.7)^E^	10.9 (5.8–16.0)^a^^E^	12.4 (8.3–16.5)^a^^E^	14.0 (7.4–20.7)^a^^b^^E^
*p*-value	N/A	< 0.0001	0.0209	N/A	N/A	N/A
Indigenous identity						
First Nations	775–782	53.1 (46.6–59.6)	24.9 (19.8–29.9)	12.6 (9.4–15.8)^a^	18.8 (13.1–24.5)^a^^E^	21.6 (14.9–28.3)^a^^E^
Mtis	859–870	67.4 (61.9–72.9)	16.0 (12.2–19.8)	24.5 (9.3–39.6)^a^^E^	17.9 (13.8–22.0)^a^	39.2 (28.9–49.6)^a^
Inuk (Inuit)	46–47	66.5 (45.6–87.5)^E^	^F^	15.0 (5.3–24.8)^a^^E^	15.1 (5.4–24.8)^a^^E^	^F^
*p*-value	N/A	0.0004	0.0018	N/A	N/A	N/A
Immigration						
Non-immigrant (Canadian-born)	33 743–34 040	57.3 (56.3–58.2)	20.8 (20.1–21.5)	13.4 (12.6–14.2)^a^	17.6 (17.0–18.3)^a^	23.3 (22.1–24.4)^a^
5 years or less since immigration	732–738	46.0 (40.1–51.9)	20.0 (15.6–24.4)	12.7 (10.3–15.1)^a^	13.0 (9.8–16.2)^a^^b^	14.7 (9.5–20.0)^a^^b^^E^
6 to 10 years since immigration	703–706	46.5 (40.7–52.3)	22.3 (17.6–27.1)	12.3 (9.2–15.3)^a^	17.5 (8.1–26.9)^a^^b^^E^	13.2 (8.6–17.9)^b^^E^
More than 10 years since immigration	4561–4601	49.1 (46.8–51.4)	27.7 (25.5–29.8)	12.7 (11.1–14.3)^a^	13.6 (12.3–14.8)^b^	15.5 (13.3–17.6)^b^
*p*-value	N/A	< 0.0001	< 0.0001	N/A	N/A	N/A
Language most spoken at home						
English only	29 738–30 002	57.6 (56.6–58.7)	20.7 (19.9–21.5)	13.6 (12.8–14.4)^a^	17.5 (16.8–18.2)^a^	23.7 (22.4–25.0)^a^
French only	5873–5931	53.0 (51.1–54.8)	22.7 (21.2–24.2)	13.6 (11.8–15.4)^a^	15.7 (14.5–16.8)^a^	17.6 (15.5–19.7)^b^
English and French	1020–1030	59.6 (54.7–64.4)	17.0 (13.7–20.3)	12.7 (10.2–15.3)^a^	18.1 (15.5–20.7)^a^	16.9 (12.3–21.5)^a^^b^
English and/or French, and other language	1988–1999	47.8 (44.3–51.3)	27.5 (24.2–30.8)	10.9 (8.8–12.9)^a^	14.4 (12.6–16.2)^a^	19.0 (15.0–23.0)^a^^b^
Other language only	1675–1690	44.4 (40.8–47.9)	27.4 (24.3–30.4)	13.3 (10.8–15.8)^a^	13.8 (9.8–17.8)^a^	12.2 (9.4–14.9)^b^
*p*-value	N/A	< 0.0001	< 0.0001	N/A	N/A	N/A
Household income						
Quintile 1 (lowest)	8990–9089	43.3 (41.4–45.2)	31.4 (29.7–33.1)	13.3 (12.0–14.5)^a^	10.4 (9.4–11.3)^a^	16.5 (14.5–18.5)^a^
Quintile 2	8475–8556	50.8 (48.7–52.8)	24.2 (22.5–25.8)	14.9 (12.8–16.9)^a^	13.9 (12.6–15.1)^b^	23.5 (20.7–26.4)^b^
Quintile 3	8120–8183	55.7 (53.7–57.6)	22.3 (20.6–23.9)	12.8 (11.5–14.0)^a^	16.5 (15.2–17.7)b,^c^	23.0 (20.6–25.4)^b^
Quintile 4	7384–7445	59.0 (56.9–61.0)	18.4 (16.8–20.0)	12.3 (10.9–13.8)^a^	19.0 (17.8–20.2)^c^^d^	19.3 (17.4–21.3)^a^^b^
Quintile 5 (highest)	7461–7521	63.8 (62.0–65.6)	15.7 (14.3–17.1)	13.1 (11.9–14.3)^a^	22.8 (20.7–24.9)^d^	22.0 (19.9–24.0)^a^^b^
*p*-value	N/A	< 0.0001	< 0.0001	N/A	N/A	N/A
Highest household education						
Less than secondary school graduation	3124–3169	32.2 (29.2–35.2)	45.5 (43.4–49.7)	11.2 (8.5–13.9)^a^	6.9 (5.3–8.6)^a^	14.8 (11.3–18.3)^a^
Secondary school graduation, no post-secondary education	6290–6362	46.1 (43.8–48.4)	31.0 (28.9–33.2)	12.1 (10.4–13.8)^a^	12.4 (11.0–13.9)^b^	21.3 (18.9–23.7)^a^
Post-secondary certificate diploma or university degree	30 486–30 707	56.9 (55.9–57.9)	19.8 (19.1–20.6)	13.4 (12.7–14.1)^a^	17.4 (16.8–18.0)^c^	21.0 (19.9–22.1)^a^
*p*-value	N/A	< 0.0001	< 0.0001	N/A	N/A	N/A
Rural/urban geography						
Rural area (less than 1000)	11 576–11 702	56.4 (54.9–57.9)	23.1 (21.9–24.3)	11.6 (10.3–12.9)^a^	17.6 (16.3–18.8)^a^	29.9 (27.8–32.0)^a^
Small population centre (1000–29 999)	8513–8596	53.9 (52.1–55.8)	23.7 (21.1–25.2)	11.7 (10.6–12.7)^a^	16.6 (15.3–17.9)^a^	23.0 (20.5–25.5)^b^
Medium population centre (30 000–99 999)	4982–5020	52.7 (50.5–55.0)	23.0 (21.0–24.9)	11.9 (10.1–13.7)^a^	17.5 (15.9–19.0)^a^	23.3 (20.2–26.3)^a^^b^
Large urban population centre (≥ 100 000)	15 359–15 476	54.3 (53.1–55.6)	21.8 (20.8–22.9)	14.3 (13.3–15.2)^a^	16.0 (15.1–17.0)^a^	17.5 (16.2–18.9)^b^
*p*-value	N/A	0.0604	0.1432	N/A	N/A	N/A
Living arrangement						
Unattached/ Single	13 871–14 028	51.0 (49.4–52.6)	26.2 (24.9–27.5)	14.9 (13.6–16.1)^a^	14.3 (13.3–15.4)^a^	18.3 (16.4–20.2)^a^
Living with spouse/partner with no children	13 919–14 042	53.5 (52.1–54.9)	24.8 (23.6–26.0)	11.8 (11.1–12.6)^b^	16.7 (15.8–17.5)^a^	19.9 (18.3–21.5)^a^
Parent living with spouse/partner and children	7090–7134	57.5 (55.7–59.4)	18.3 (16.9–19.7)	13.0 (11.7–14.4)^a^^b^	16.4 (15.4–17.3)^a^	23.1 (21.0–25.3)^a^
Single parent living with children	1883–1901	51.7 (48.0–55.4)	27.7 (24.2–31.1)	10.8 (8.7–13.0)^a^^b^	14.0 (12.3–15.6)^a^	21.2 (16.9–25.6)^a^
*p*-value	N/A	< 0.0001	< 0.0001	N/A	N/A	N/A
Sexual orientation						
Heterosexual	37 362–37 679	55.2 (54.4–56.1)	21.7 (21.0–22.5)	13.3 (12.6–14.0)^a^	16.6 (16.1–17.2)^a^	21.4 (20.4–22.4)^a^
Gay or lesbian	564–566	61.2 (54.6–67.9)	16.9 (11.9–21.9)^E^	19.4 (13.8–25.1)^a^	17.9 (14.4–21.4)^a^	17.0 (11.4–22.6)^a^^E^
Bisexual or pansexual	639–645	65.1 (58.7–71.5)	9.1 (5.4–12.8)^E^	16.5 (13.2–19.8)^a^	25.7 (11.9, 39.4)^a^^E^	20.7 (14.5–27.0)^a^^E^
*p*-value	N/A	0.0029	< 0.0001	N/A	N/A	N/A
Age groups						
18–34 years	6042–6081	59.6 (57.8–61.5)	15.6 (14.1–17.0)	14.1 (12.7–15.6)^a^	20.1 (18.3–21.9)^a^	22.0 (20.0–24.1)^a^
35–49 years	7829–7880	58.4 (56.6–60.2)	16.6 (15.3–17.9)	13.3 (11.9–14.8)^a^	16.9 (15.9–17.9)^a^	26.4 (23.9–28.8)^a^
50–64 years	10 140–10 233	57.0 (55.2–58.7)	22.9 (21.4–24.4)	14.6 (13.2–15.9)^a^	16.2 (15.1–17.2)^a^^b^	21.0 (19.1–23.0)^a^
65–79 years	12 896–13 025	45.6 (44.1–47.0)	31.8 (30.5–33.2)	11.6 (10.8–12.3)^a^	13.7 (12.7–14.6)^b^	14.8 (13.4–16.1)^b^
80 years and older	3523–3571	24.4 (21.1–26.8)	54.3 (51.4–57.2)	7.2 (5.8–8.6)^b^	5.6 (4.7–6.5)^c^	7.3 (5.0–9.6)^c^^E^
*p*-value	N/A	< 0.0001	< 0.0001	N/A	N/A	N/A
Living with chronic condition(s)d						
No chronic conditions	20 520–20 666	58.1 (57.0–59.2)	18.4 (17.4–19.3)	14.3 (13.3–15.2)^a^	18.5 (17.6–19.4)^a^	21.1 (19.9–22.4)^a^
One chronic condition	13 247–13 389	52.7 (51.1–54.2)	24.7 (23.4–26.0)	12.6 (11.6–13.7)^a^	14.7 (13.8–15.6)^b^	21.9 (19.9–24.0)^b^
Multimorbidity	6655–6727	38.5 (36.3–40.7)	39.5 (37.3–41.7)	9.1 (7.9–10.3)^b^	9.7 (8.5–10.8)^c^	16.5 (14.1–18.9)^c^
*p*-value	N/A	< 0.0001	< 0.0001	N/A	N/A	N/A
Employment status (aged 18 to 75 years)						
Employed full-time	16 147-16 301	60.5 (59.2–61.8)	17.0 (16.1–18.0)	14.0 (12.9–15.1)^a^	17.9 (16.9–18.9)^a^	27.8 (26.1–29.5)^a^
Employed part-time	3020-3056	57.0 (53.8–60.2)	16.2 (13.7–18.6)	15.2 (12.8–17.6)^a^	17.5 (15.6–19.4)^a^	19.4 (15.5–23.3)^a^,b
Unemployed	14 898–15 028	50.1 (48.5–51.6)	26.1 (24.8–27.4)	12.9 (12.0–13.8)^a^	15.9 (14.9–16.8)^a^	13.2 (12.1–14.2)^b^
*p*-value	N/A	< 0.0001	< 0.0001	N/A	N/A	N/A

Source: Canadian Community Health Survey 2021. 

Abbreviations: CI, confidence interval; CV, coefficient of variation; MVPA, moderate-to-vigorous intensity physical activity; PA, physical activity. 

Notes: p-values were calculated using chi-square tests. Means with different superscripts (a or b or c) indicate that the means were statistically significantly different at p ≤ 0.001 based on Tukey-Kramer post-hoc comparisons (i.e. the same superscript letter indicates that the means were not statistically different from each other). 

d Chronic conditions included diabetes, arthritis, heart disease, anxiety (e.g. phobia, obsessive-compulsive disorder) or depressive (e.g. depression, bipolar, mania, dysthymia) disorders, cancer, Alzheimer’s, or the effects of a stroke. 

E There is high sampling variability associated with these estimates (i.e. 15 < CV ≤ 35). 

F These estimates do not meet Statistics Canada’s quality standards and therefore cannot be reported (i.e. CV > 35). 

## Discussion

Using a large national sample of individuals living in Canada, we sought to explore differences in self-reported PA across population subgroups using a health equity lens among youth and adults. Our results identified inequalities in PA across several subgroups. A substantially lower percentage of girls and women were meeting PA recommendations, and girls and women reported fewer minutes of PA across most domains compared to boys and men. The percentage of girls meeting the PA recommendation was nearly 20 percentage points lower than in boys, with narrowing but significant differences persisting into adulthood. Among youth and adults, we found an income and education gradient where more advantaged subgroups reported significantly more recreation PA than their less advantaged counterparts. Active transportation did not differ across population subgroups for youth and adults. 

When comparing our results with data using objective measures (i.e. accelerometers), we found a few inconsistencies. Colley et al.^20^ found that adults without a spouse or children had the highest percentage of meeting the PA recommendations. In contrast, our results indicate that unattached/single adults had the lowest percentage of meeting the PA recommendations when compared with all other living arrangements. This divergence of results could be due to a self-report bias, although it is difficult to fully understand why these differences occurred. However, there were several consistencies between our findings versus the PASS Indicators^5^ and Colley et al.^20^ device measured results among adults, including a decreasing gradient in meeting recommendations with age, higher percentage of meeting PA recommendations among males compared to females, and an increasing gradient in meeting PA recommendations with higher education levels. In addition, our results for youth are consistent with the PASS Indicators’ device measured results that show higher percentage of meeting PA recommendations among males compared to females aged 5-17 years.

Large sex and gender inequalities in PA remain a concern in Canada, which are congruent with other research.^28^,^29^ Our results demonstrated a larger significant gender gap in meeting PA recommendations among youth, with the gap narrowing but remaining significantly different between men and women in adulthood. Our data suggest that the narrowed gender gap in PA could be driven by a large change in recreational PA among adults when compared to youth, something that should be investigated further. Some studies have identified higher income inequality within a country being associated with a larger gender gap in PA,^30^ suggesting that there are potential structural issues and unequal access to PA that could explain these inequalities. Policies and interventions to address gender inequalities remain an important area of future research. 

We found inequalities in household income and household education categories for recreation PA among both youth and adults; however, these findings differed from previous research. For instance, in Chile^16^ and Germany^17^ those in the lowest income and education categories reported the most PA compared to those in the highest categories. This was inverse to our findings; however, in the German study^17^ PA was measured using the Global Physical Activity Questionnaire which measured occupational PA differently than our PA questionnaire. Thus, differences in the PA measurement tools may explain these divergent findings. 

Our study highlights a few unexpected results that contrast with previous findings. For instance, although we observed small significant differences in meeting PA recommendations and no MVPA across sexual orientation categories for adults, there were no significant differences across PA domains. These results do not align with other national estimates that indicate higher PA levels among lesbian or gay groups.^31^ Our divergent findings might represent a lack of sample size to identify differences, or it could indicate that there are other intersecting factors involved. For instance, one study identified that among the gay or lesbian group, both gender and income were important for identifying individuals with a higher likelihood of being physically active.^32^ It is possible that taking an intersectional approach to PA would help better identify subgroups within the gay or lesbian group (e.g. by ethnicity, education, income, gender, health status) that report lower levels of PA. Although we did not apply an intersectional approach, we provide a broad overview of factors that may help inform intervention targets and guide future intersectional research in this area. 

There was a lack of significant differences in self-reported active transportation within most population subgroups. Although an important aspect of health equity research is to find inequalities to help guide targeted interventions and policy, we believe that the absence of inequalities in active transportation, in itself, is an important finding. This may suggest that efforts to promote active transportation through policies, improved and accessible built environments, and interventions might be an equitable approach to increasing population PA. Active transportation has shown to be an important domain for increasing the likelihood of adherence to the physical activity recommendations.^33^ In Canada, substantial work is underway to promote active transportation, including standardized nomenclature around comfort and safety,^34^ inventories of available infrastructure,^35^ evaluations of the impacts of new infrastructure,^36^,^37^ and investments in infrastructure through the National Active Transportation Strategy.^38^ Continued research efforts in this area are important as they may have implications for supporting equitable increases in population PA.

***Strengths and limitations***

There are several strengths to this research, including the large national sample, data for several domains of PA, and investigation of differences across a wide range of population groups in a sample of both youth and adults. Despite these strengths, there were several weaknesses that should be considered. First, the sample size for several under-represented subgroups (e.g. gender diverse, Indigenous identity) was small, making it impossible to identify potential differences. Statistics Canada’s Disaggregated Data Action Plan was developed to address some of these data limitations.^39^ Second, although we were able to examine differences across many population subgroups, there were some important subgroups missing from our analysis, such as participants living with cognitive, behavioural or physical functional difficulties. Third, the 2021 CCHS adult PA questions asked to report PA accrued in bouts of 10 minutes or more; this is no longer part of the new PA recommendation which recognizes the benefit of every minute of PA.^4^ Previous research in Canada suggests that, although the removal of the 10-minute bout requirement would increase the number of individuals meeting the recommendation, there are no differences in the demographic, behaviour or health profiles of those captured by the new recommendation.^40^ Fourth, there is a potential for self-report bias and for this bias to vary between population groups. For instance, a systematic review that compared self-report to device measured PA found a greater bias in women compared to men.^41^ Fifth, the data were collected during the COVID-19 pandemic, which may have contributed to the low response rates. Canadian research has shown that PA levels of youth and men aged 18-64 years dropped during the pandemic compared to before the pandemic, particularly among girls.^42^,^43^ Among youth there was also a substantial drop in transportation and recreation PA, whereas recreation PA increased in adults.^42^ Given the potential effects of the pandemic on PA, future work would need to assess whether the differences observed in this study remain consistent or if there is a further widening or narrowing of inequalities in PA. Lastly, our analysis explored individual subgroups independent of other factors. People do not exist in a single stratum and future work would need to further explore intersections among the population. There have been large advancements in intersectional health research using decision tree, cluster analyses, and person-centred techniques to identify combinations of population subgroup characteristics.^13^,^20^ Use of these methods may be a valuable means to further explore heterogeneity and inequalities in PA based on these intersections. 

## Conclusion

Significant inequalities in PA exist in Canada. The largest inequalities in PA were observed by sex and gender, household income, and highest household education. There is a need to explore ways to improve equality in recreation PA, as this was the domain with the most significant differences across population subgroups. Active transportation had few differences across subgroups, representing a potential avenue for equitable intervention to increase population PA levels. Future research should explore why these inequalities exist to help inform interventions. 

## Acknowledgements

This study received no funding.

## Conflicts of interest

Justin J. Lang is the journal’s Associate Editor-in-Chief and also one of the Associate Scientific Editors, but has recused himself from the review process for this article.

The authors have declared no conflicts of interest.

## Authors’ contributions and statement

JJL: Conceptualization, data curation, formal analysis, investigation, methodology, validation, writing – original draft.

SET: Formal analysis, methodology, validation, writing – review and editing.

ND: Investigation, writing – review and editing.

ID: Investigation, writing – review and editing.

SAP: Investigation, writing – review and editing.

KCR: Investigation, writing – review and editing.

MTB: Investigation, writing – review and editing.

RCC: Investigation, writing – review and editing.

RJ: Investigation, writing – review and editing.

AA: Investigation, writing – review and editing.

JPC: Conceptualization, investigation, writing– review and editing.

The content and views expressed in this article are those of the authors and do not necessarily reflect those of the Government of Canada.
